# Clinical Profile and Management of Rheumatic Heart Disease in Children and Young Adults at a Tertiary Cardiac Center in Indonesia

**DOI:** 10.3389/fsurg.2020.00047

**Published:** 2020-08-12

**Authors:** Oktavia Lilyasari, Radityo Prakoso, Yovi Kurniawati, Poppy S. Roebiono, Anna Ulfah Rahajoe, Indriwanto Sakidjan, Ganesja M. Harimurti

**Affiliations:** Department of Cardiology and Vascular Medicine, Faculty of Medicine Universitas Indonesia, National Cardiovascular Centre Harapan Kita, Jakarta, Indonesia

**Keywords:** rheumatic heart disease, rheumatic reactivation, valvular lesion, valve surgery, children, young adult

## Abstract

**Introduction:** Rheumatic heart disease (RHD) remains a major public health issue affecting children and young adults in developing countries. This study aimed to evaluate the clinical characteristics, management, and reactivation of RHD among children and young adults.

**Patients and Methods:** This was a hospital-based retrospective study conducted at the National Cardiovascular Center Harapan Kita, Indonesia; we retrieved relevant data from patients diagnosed with RHD between 2012 and 2018.

**Results:** Two hundred and seventy-nine patients were diagnosed with rheumatic heart disease, of whom 108 were children (mean age of 12.02 ± 3.36 years) and 171 were young adults (mean age was 24.9 ± 3.84). RHD was more common in female than in male young adults (1.5:1). Hospitalization due to RHD complications such as congestive heart failure was seen in 11.11% of cases in children, while pulmonary hypertension was present in 19.95% young adult cases. Reactivation of RHD occurred in 17.2% (48/279) cases, significantly in children (*p* < 0.001). Overall, the mitral valve (either isolated or combined) was the organ most affected in children (39.13%) and young adults (44.81%). Isolated mitral regurgitation was more common in children (13/21, 61.9%), while isolated mitral stenosis was more common in young adults (19/47, 40.42%). There was a high rate of rheumatic tricuspid valve disease in all populations (193/279, 69.17%) and reported involvement of pulmonary regurgitation (46/279, 16.48%). Multivalve lesions were more common than single lesions in both groups, with a combination of mitral and tricuspid regurgitation the predominant type in children (32/43, 74.41%) and mixed mitral lesion and tricuspid regurgitation in young adults (22/72, 30.56%). We observed a significant occurrence of quadrivalve lesions in children (*p* = 0.039). Valve repair was more common in children (49.07%) and replacement in young adults (32.16%), with low in-hospital mortality. Compliance with secondary prophylaxis was a significant challenge.

**Conclusion:** Chronic RHD often presented with complications of the disease or reactivation of rheumatic fever (RF). Inadequate treatment of RF/RHD leads to extensive valvular damage and consequent disabilities. Efforts toward active early diagnosis and prompt treatment of RF/RHD and effective preventive measures are essential.

## Introduction

Rheumatic heart disease (RHD) remains a major public health problem, accounting for 320,000 deaths and around 10.5 million people with disabilities globally (2015) (1). Although RHD is preventable, underestimation of its prevalence leads to a neglect of primary prevention in national health policies and eventual progressive occurrence and fatality in children and young adults, especially in developing countries (1, 2).

Rheumatic fever (RF) usually affects school-age children, with preceding infection of group A streptococcus (GAS) that manifests in multiple symptoms. Risk factors of streptococcal infection include poverty, malnutrition, overcrowding, poor housing, and shortage of healthcare resources, underlining the high prevalence in developing countries (1, 3). All manifestations of RF resolve completely after, except for cardiac valvular lesion, which is the hallmark of RHD. Postrheumatic valvulopathies remain the main cause of heart failure among children and young adults (3). Therefore, patients with clinical features of RF/RHD should be promptly treated and referred for definite diagnosis and long-term management to limit the extent of heart damage. However, in developing countries where access to medical care is difficult, patients often present to tertiary care with already disabling symptoms such as congestive heart failure or pulmonary hypertension (2, 3). Secondary penicillin prophylaxis to prevent reactivation of RHD should be undertaken by all patients, but long-term management, and patient compliance have been challenging (4).

We sought to determine the clinical characteristic and pattern of RHD among children and young adults, conveying any clinical importance, and comprehensive understanding of RHD that may aid future diagnosis and management. The study also had the purpose of raising practicing clinicians' and governments' awareness of RHD and its clinical burden.

## Patients and Methods

We retrospectively analyzed the clinical characteristics of patients with RHD registered at National Cardiovascular Center Harapan Kita, as the tertiary referral cardiovascular center in Indonesia between 2012 and 2018. The study was reviewed and approved by the Ethical Committee of Department of Cardiology and Vascular Medicine, Faculty of Medicine Universitas Indonesia, National Cardiovascular Center Harapan Kita. The study obtained data regarding 108 children, ages 3–18 years old, and 171 young adults, ages >18–30 years old, when diagnosed with RHD. Information obtained from the records included age, gender, clinical symptoms, laboratory and echocardiography reports, and management decisions. Clinical and physical examinations were carried out by cardiologists to determine patients' symptoms and conditions. An electrocardiogram was performed on all subjects to determine valvular involvement and other accompanying conditions (e.g., atrial fibrillation). Echocardiography was obtained by cardiologists and pediatric cardiologists for adult and child patients to determine abnormalities, especially valvular damage. Diagnosis of RHD was based on the previous history of acute RF with valvular lesion, established diagnosis of RHD at previous centers, and/or the World Heart Federation criteria for echocardiographic diagnosis of RHD at time of admission to our center. Reactivation of RHD was defined as patients who had two major or one major and two minor or three minor of the revised Jones criteria for high-risk populations with a history of previous RF or previous valvular involvement of rheumatic origin by the time of enrollment. Several subjects suspected with recent upper respiratory tract infection were examined with throat swab culture. Following the diagnosis, patients underwent treatment suitable for each case and were followed up until discharge. Any readmission due to reactivation of RHD, postsurgical complications, worsening symptoms, or related disease were noted. Data were recorded and analyzed using the IBM SPSS statistic version 23.

## Results

During the 7 years of the study period, 108 children were diagnosed with RHD; the mean age was 12.02 ± 3.36 years, with a male to female ratio of 1:1. Meanwhile, of the 171 young adults admitted with RHD, the mean age was 24.9 ± 3.84; 39.18% were male and 60.81% female. Baseline characteristics of RHD patients are described in Table 1.

**Table 1:** Baseline characteristics of RHD patients.

**Characteristics**	**Children (*n* = 108)**	**Young adults (*n* = 171)**
Gender		
Female	54 (50%)	104 (60.81%)
Male	54 (50%)	67 (39.18%)
Age		
Mean	12.02 ± 3.36	24.9 ± 3.84
Range	3–17	>18–30

### Clinical Presentation and Reactivation of RHD at Time of Admission

Almost all patients, children and young adult, had cardiovascular symptoms during admission (dyspnea, peripheral edema, syncope, palpitation, or chest pain), some (48/279, 17.2%) presented with symptoms of reactivation (carditis, fever, etc.), and the rest were referred for surgical management without significant symptoms (Table 2). In the children's group, patients were hospitalized due to severe congestive heart failure (12/108, 11.11%), but in the young adult group patients were more commonly admitted due to severe pulmonary hypertension (29/171, 19.95%). Atrial fibrillation was more often seen in young adults (37/171, 21.63%) than in children (5/108, 4.62%). Other reasons for hospitalization in children included pericardial effusion, bronchopneumonia, infective endocarditis, and pneumothorax. Meanwhile in young adults, pericardial effusion and pneumonia had been reported in addition to RHD.

**Table 2:** Clinical manifestations and laboratory investigation findings in RHD cases.

**Clinical manifestations**	**Children** **(*n* = 108)**	**Young adults** **(*n* = 171)**	**Total** **(*n* = 279)**
Clinical presentation			
Pulmonary hypertensions	11 (10.18%)	29 (19.95%)	40
Congestive heart failure	12 (11.11%)	11 (6.43%)	23
Atrial fibrillation	5 (4.62%)	37 (21.63%)	42
Pericardial effusion	2 (1.85%)	1 (0.58%)	3
Pneumonia	4 (3.70%)	1 (0.58%)	5
Infective endocarditis	4 (3.70%)	—	4
Major criteria			
Carditis	72 (66.67%)	106 (61.98%)	178
Arthritis	28 (25.92%)	8 (4.68%)	36
Chorea	0	2 (1.16%)	2
Subcutaneous nodules	5 (4.62%)	2 (1.16%)	7
Erythema marginatum	6 (5.56%)	2 (1.16%)	8
Minor criteria			
Arthralgia	27 (25%)	6 (3.51%)	33
Fever	45 (41.67%)	21 (12.28%)	66
Elevated C-reactive protein	49 (45.37%)	10 (9.26%)	59
Elevated erythrocyte sedimentation rate	42 (38.89%)	23 (13.45%)	65
Prolonged PR interval	4 (3.70%)	2 (1.16%)	6
Leucocytosis	53 (49.07%)	47 (27.49%)	100
Suggesting evidence of preceding infection			
Elevated ASTO	26 (24.07%)	6 (3.51%)	32
Positive throat culture	18 (16.67%)	3 (1.75%)	21

Most patients had carditis at admission: 72 children (66.67%) and 106 young adults (61.98%) (*p* = 0.428). Some children experienced arthritis (28/108, 25.92%) compared to fewer cases in young adults. Among others, arthralgia (27/108, 25%), fever (45/108, 41.67%), elevated C-reactive protein (49/108, 45.37%), and elevated erythrocyte sedimentation rate (42/108, 38.89%) were significantly more common in children (*p* < 0.000). Prolonged PR intervals (4/108, 3.7%) were also more commonly seen in children than in young adults (*p* = 0.155) (Table 2).

Patients who were diagnosed with reactivation of RHD at time of admission already had an established diagnosis of RHD from previous hospitals and were referred to our center when reactivation occurred. Reactivation of RHD was noted to be more common in children (39/108, 36.1%) than in young adults (9/171, 5.26%) (*p* < 0.001), with carditis as the most common major criteria reported in both children (23/39, 58.97%), and in all (9) young adult patients. Arthritis (6/9, 66.67%), chorea (2/9, 22.22%), subcutaneous nodules (2/9, 22.22%), and erythema marginatum (2/9, 22.22%) were more commonly seen in reactivation cases of young adults (*p* = 0.404, *p* = 0.003, *p* = 0.198, *p* = 0.328, respectively). No report of chorea was reported in the children's group. Like the major criteria, arthralgia (5/9, 55.56%) and fever (7/9, 77.78%) were more common in young adult cases compared to children (*p* = 0.348, *p* = 0.359, respectively). Among these reactivation cases, 30 (62.5%) patients had elevated antistreptolysin-O (ASTO) (26 of these were reported in the children's group), and 17 (35.41%) patients had a positive throat culture (16 from the children's group). All patients with reactivation of RHD were hospitalized and treated with an eradication dose of phenoxymethylpenicillin (PMP) and steroids.

### Valve Involvement

Mitral valve involvement was the most commonly affected valve in both children and young adults, followed by tricuspid, aortic, and pulmonary valves. Of 99 (39.13%) children with mitral involvement, 93 had mitral regurgitation (93.93%), 2 had stenosis (2.02%), and 4 had both regurgitation and stenosis (4.04%). In contrast, mitral stenosis was slightly more common in adults (57/160, 35.62%), followed by mitral regurgitation (53/160, 33.12%) and both regurgitation and stenosis lesions (50/160, 31.25%). Meanwhile, only tricuspid regurgitation was reported in children (80/108, 31.62%), as well as aortic regurgitation (53/108, 20.94%). Pulmonary regurgitation was the least reported in both groups (Table 3).

**Table 3:** Valvular affectation in RHD patients.

**Characteristics**	**Children**	**Young adults**	**Total**	***p*-value**
Mitral valve lesions	99 (39.13%)	160 (44.81%)	259	0.383
Overall MR	93	53	145	
Overall MS	2	57	59	
Overall MS + MR	4	50	54	
Tricuspid valve lesions	80 (31.62%)	113 (31.65%)	193	0.159
Overall TR	80	113	193	
Aortic valve lesions	53 (20.94%)	59 (16.52%)	112	0.016
Overall AR	53	50	103	
Overall AS	—	2	2	
Overall AS + AR	—	7	7	
Pulmonary valve lesions	21 (8.3%)	25 (7%)	46	0.211
Overall PR	21	25	46	
Total	253	357	610	

Single-valve lesions in both groups presented as isolated mitral lesions, with isolated MR in most children (13/21, 61.9%) (*p* = 0.002) and isolated MS in most adults (19/47, 40.42%) (*p* = 0.003). Isolated tricuspid regurgitation was reported only in children, while isolated mixed aortic lesion (AS + AR) was reported only in young adults. There was no report of isolated pulmonary lesions in any population, albeit they always appeared in combination with others. Frequencies of multivalvular lesions were much higher than those of isolated single-valve disease in both age groups (*p* = 0.128). As seen in Table 4, dual-valve lesion was more common in children (43/108, 39.81%) than in young adults (72/171, 42.11%) (*p* = 0.705). It was followed by triple-valve (30/108, 28.70%), single-valve (21/108, 19.40%), and quadrivalve (14/108, 12.96%) lesions. In comparison, the single-valve lesion was the next common lesion (47/171, 27.48%) in the young adult group, followed by triple-valve (42/171, 4.56%) and lastly all-valve (10/171, 5.84%) lesions. The most common dual-valve lesion was the combination of mitral regurgitation (MR) and tricuspid regurgitation (TR) in children (32/43, 74.41%) (*p* < 0.001), while in young adults mixed mitral valve (MR + MS) lesion and tricuspid regurgitation was more common (28/72, 38.9%) (*p* < 0.001). A combination of MR, TR, and AR was the most commonly seen triple-valve disease in children (23/30, 76.67%) and young adults (27/42, 64.28%) (Table 4). Quadrivalve lesion was significantly more common in the children's group (14/108, 12.96%) than in young adults (10/171, 5.84%) (*p* = 0.039) (Table 4) and often presented as a combination of MR + TR + AR + PR in both groups.

**Table 4:** Pattern of valve involvement and type of lesions in RHD patients.

**Characteristics**	**Children** **(*n* = 108)**	**Young adults** **(*n* = 171)**	**Total** **(*n* = 279)**
Single valve lesion	21 (19.40%)	47 (27.48%)	68
Isolated mitral	14 (12.96%)	38 (22.22%)	52
Isolated MR	13 (12.03%)	11 (6.43%)	24
Isolated MS	1 (0.92%)	19 (11.11%)	20
Isolated MS + MR	—	8 (4.67%)	8
Isolated tricuspid	2	—	2
Isolated TR	2 (1.85%)	—	2
Isolated aorta	5	9 (5.26%)	14
Isolated AR	5 (4.62%)	7 (4.09%)	12
Isolated AS + AR	—	2 (1.16%)	2
Multivalve lesion			
Dual valve	43 (39.81%)	72 (42.11%)	115
M + T	33 (30.55%)	60 (35.08%)	93
MR + TR	32 (29.62%)	10 (5.84%)	41
MS + TR	—	22 (12.86%)	23
MS + MR + TR	1 (0.92%)	28 (16.37%)	28
M + A	8	9 (5.26%)	17
MR + AR	8 (7.41%)	7 (4.09%)	15
MR + AS + AR	—	1 (0.58%)	1
MS + AS + AR		1 (0.58%)	1
M + P	—	1	1
MS + MR + PR	—	1 (0.58%)	1
T + A	2	2 (1.2%)	4
TR + AR	2 (1.85%)	1 (0.58%)	3
TR + AS	—	1 (0.58%)	1
Triple valve	30 (27.78%)	42 (24.56%)	72
M + T + A	23	27 (15.78%)	50
MR + TR + AR	23 (21.29%)	12 (7.01%)	35
MS + TR + AR	—	6 (3.51%)	6
MS + TR + AS + AR	—	1 (0.58%)	1
MS + MR + TR + AR	—	8 (4.67%)	8
M + T + P	6 (5.56%)	14 (8.18%)	20
MR + TR + PR	3 (2.78%)	5 (2.92%)	7
MS + TR + PR	1 (0.92%)	5 (2.92%)	6
MS + MR + TR + PR	2 (1.85%)	4 (2.39%)	6
M + A + P	1	1	2
MR + AR + PR	1 (0.92%)	1 (0.58%)	2
All valves	14 (12.96%)	10 (5.84%)	24
MR + TR + AR + PR	13 (12.03%)	3 (1.75%)	16
MR + TR + AS + AR + PR	—	1 (0.58%)	1
MS + TR + AR + PR	—	3 (1.75%)	3
MS + TR + AS + PR	—	1 (0.58%)	1
MS + TR + AS + AR + PR	—	1 (0.58%)	1
MS + MR + TR + AR + PR	1 (0.92%)	1 (0.58%)	2

### Management

In both groups, surgery was more often performed, with the rest undergoing either percutaneous balloon mitral valvuloplasty (BMV) or only medically managed (Table 5). Of 108 children patients, 67 (62.03%) had received surgical intervention, of whom 53 (79.1%) had valve repairment, 9 (13.43%) had valve replacement, and 5 (7.46%) had both valve replacement and repair. There was no reported postoperative or in-hospital mortality in the children's group. Of 171 young adults, 98 (57.3%) had surgery; 55 (56.12%) had valve replacement, 28 (28.57) had valve repair, and 15 (15.3%) had both repair and replacement. Two young adult patients died during hospitalization. The first case was a 21-year-old female with severe mitral regurgitation and tricuspid regurgitation who underwent mitral valve replacement procedure and died a day after due to critical pulmonary hypertension (PH). The second was a 23-year-old female with severe mitral regurgitation and stenosis who underwent urgent BMV; however, the procedure failed due to the patient's current condition and the patient died due to critical PH and intractable heart failure.

**Table 5:** Management of rheumatic heart disease.

**Intervention**	**Children** **(*n* = 108)**	**Young adults** **(*n* = 171)**	**Total** **(*n* = 279)**
Surgery	67 (62.03%)	98 (57.30%)	165 (59.13%)
Valve replacement	53 (49.07%)	28 (16.37%)	81 (47.36%)
Valve repairment	9 (8.33%)	55 (32.16%)	64 (37.42%)
Valve replacement and repairment	5 (4.62%)	15 (8.77%)	29 (16.95%)
BMV	2 (1.85%)	45 (26.31%)	47 (27.48%)

Two patients had been in their last trimester of pregnancy during the time of diagnosis with presenting symptoms of dyspnea and peripheral edema. Both had required hospitalization during pregnancy and underwent selective C-section due to their present conditions. The patients were then treated for mitral stenosis due to RHD, with one patient eventually undergoing BMV with a satisfactory result.

### Follow-Up and Readmission

Children had an average length of stay of 12.74 days, which was longer than the average stay of young adults, which was 6.82 days. Patients were followed up until discharge and were given secondary prophylaxis. All patients were advised to practice routine control measures in 6 months to 1 year if possible once deemed appropriate by attending cardiologists. However, most patients were referred back to their previous hospital, where cardiologist and echocardiography evaluation were available. Among all cases of RHD, only 20 patients (7.16%) were readmitted to our hospital during the period of study for various reasons; two were readmitted more than once. Generally, patients were rehospitalized for a surgical or valvuloplasty procedure. Three patients returned due to symptoms of congestive heart failure; one child patient with a post valve repair procedure developed reactivation of RHD 4 years later although she had been treated with secondary prophylaxis for a 1-year period. Two complained of palpitations caused by atrial fibrillation; one came back due to anticoagulant poisoning after treatment of atrial fibrillation. One child patient had clinical manifestation of carditis, fever, and arthralgia but no evidence of preceding streptococcal infection. Readmission was noted to be more common in young adults (60%) than in children (40%).

## Discussion

Rheumatic heart disease (RHD) is an eminently preventable chronic cardiac disease, and its prevention lies on the understanding of clinical manifestations for timely diagnosis and prompt treatment as per regimen. The patients in this study were predominantly young adults with advanced RHD, supporting the suggestion that rheumatic valvular abnormalities would be advancing into adulthood. The mean age on admission of children with RHD patients was 12.02 ± 3.36, consistent with school-age children being at high risk (2), and 24.9 ± 3.84 for young adults. In the children's group, both sexes were equally affected by the disease, yet there was a slight predominance of RHD in female young adults than in males, with a male to female ratio 1:1.5. This is particularly similar to previous RHD studies in which the disease was more prevalent in females than in males (2, 5, 6). However, there had been reports of a male predominance in Lagos (7). No study clearly identified significant reasons for young adult female predominance in RHD; whether this was a result of innate susceptibility in child-rearing age or cultural and social positions of women is unclear (8).

### Clinical Manifestations and Reactivation of RHD in Children and Young Adults

RHD is the leading of cause of death of cardiac origin (9). More often than not, patients with RHD presented at hospital with worsening symptoms due to complications of RHD. Infective endocarditis, embolic event, atrial fibrillation, heart failure, and pulmonary hypertension were some of the most common complications of untreated severe rheumatic valvular disease (10). Distinguished complications of advanced RHD, particularly congestive heart failure and pulmonary hypertension, were all seen commonly in both children and young adults. Forty (14.33%) patients, of whom 11 were children and 29 were young adults, had secondary pulmonary hypertension, and almost half of these were severe. Pulmonary hypertension develops early and is a usual occurrence in patients with valvular heart disease, particularly in mitral and aortic valve lesions, where the left ventricle (LV) becomes hypertrophic and less distensible, leading to increased LV end-diastolic pressure. As a result, the left atrium (LA) also becomes hypertrophic and enlarged, reducing its output and then pulmonary hypertension ensues (8). Atrial fibrillation occurred more often in the young adult population and was particularly significant given its debilitating complication of spontaneous thrombosis LA, along with limited access to anticoagulants in rural areas (1, 8). Infective endocarditis (IE) was also detected in the child population, which was in accordance with a previous study in which IE was observed in 16% children with RHD (3). RHD was found to be the cause of 84% cases of IE, specifically in Asian countries (3, 11). This association was potentially fatal, considering that IE is challenging to diagnose and manage, especially with limited diagnostic support. These complications often became the chief complaints, not the actual rheumatic fever itself, indicating that an acute episode of RF was often unrecognized and left untreated. This signified the importance of educating the community and practicing clinicians in rural areas about RF/RHD for timely identification and prompt treatment in an effort to prevent further unwanted complications. Proper access to laboratory facilities in confirming the present of streptococcal infections is also necessary in making diagnosis.

Cardiac involvement in RHD involves the pericardium, myocardium, and endocardium, with valvulitis being the most common presentation, ranging widely from subclinical to severe cases with congestive heart failure and/or death (12). Carditis was the prevalent major criterion in both child and young adult groups and accounted for an overall 63.79% patients in this study, which was similar to other findings of carditis ranging from 37.5 to 93% (2). Polyarthritis and skin findings, subcutaneous nodules and erythema marginatum, occurred more often in children. Both skin findings were infrequent manifestations that occurred in fewer than of 10% patients in a previous study (13). Chorea was seen only in the young adult population, frequently affected the face asymmetrically, and rarely occurred as a sole manifestation. A previous study, however, had reported a prevalence of chorea in 1.4% cases among 211 children in Nepal (3). Fever was the second most common clinical manifestation in both groups, followed by a rise in inflammatory markers. Arthralgia was a more frequent complaint among children.

The revised 2015 Jones criteria for medium- and high-risk populations considered monoarthritis and polyarthralgia as major criteria. Based on the modified criteria, reactivation of RHD was significantly higher in children than in young adults (*p* < 0.001), highlighting that children are at a higher risk of recurrent RF, in contrast to a study in Bangladesh where recurrent acute RF was more commonly observed in adults (54.3%) than in children (35%) (14). In addition, supporting evidence of recent streptococcal infection (prolonged PR interval, elevated ASTO, positive throat culture) was more frequently described in the children's group.

The clinical manifestation of recurrent RHD mimics that of acute endocarditis, especially when it is the isolated manifestation, complicating the diagnostic process (15). Some studies have claimed that the Jones criteria is more applicable to the first episode of rheumatic fever, and recurrence should be suspected in any new cardiovascular manifestation in patients with chronic RHD (15). All these patients were treated with antibiotics for eradicating streptococcal infection, with the addition of steroids to suppress inflammatory responses and minimize the effects of inflammation. Administration of steroids instead of salicylates in acute RF or reactivation was more preferred in controlling the inflammatory process (2). The recommended initial dose of prednisone (1–2 mg/kg body wt/day, max. 80 mg/day) was given for 2–3 weeks, then tapering the dosage (16).

### Pattern of Valvular Involvement in RHD

Mitral valve lesion was the predominant rheumatic valvulopathy in both the children's and young adult groups. The prevalent involvement of the mitral valve had been extensively described in the literature including in a previous study at a tertiary care center in other parts of developing countries, including Nepal (3, 5, 17), India (18), and Pakistan (6), usually followed by aortic valve disease.

Regarding single valvular lesion, isolated pure mitral valve lesion (12.96%) was affected more than isolated aortic valve lesion (4.62%) in the children's population. A comparable result was seen in the young adult population, with isolated mitral valve lesion (22.22%) more prominent than isolated aortic valve lesion (5.26%). Similarly, studies done by Laudari et al. and Alkhalifa et al. both showed that isolated mitral valve lesion was the most commonly affected valve lesion (46.8 and 60%, respectively) (19, 20).

RHD predominantly affects the left-sided cardiac valves, although the reason is still unclear (21). In contrast with our study, however, rheumatic tricuspid valve disease was the second most common in all RHD populations. Our data recorded two cases of isolated tricuspid valve lesion in children, which is very rare, with most cases usually being functional or with the presence of other valve lesions. The first case was a 14-year-old male presenting with symptoms of acute RF (carditis, polyarthritis, subcutaneous nodule, and fever), and echocardiography showed moderate tricuspid regurgitation; a history of previous RF was unclear. Another case was a 10-year-old male presenting with signs of congestive heart failure (orthopnea, paroxysmal nocturnal dyspnea, dyspnea on effort), and echocardiographic examination found mild tricuspid regurgitation of rheumatic origin. These patients were then treated with secondary prophylaxis (PMP).

In our study most patients had a multivalve lesion (80.6% children and 72.52% young adults), indicating the advanced stage of the disease. The incidence was even higher than that of single valvular disease in both groups. A similar observation was reported in the study by Faheem et al. in Pakistan, where multivalvular lesions accounted for more than half (56.30%) of the overall study population (6). This may be because their study and ours were both done at a tertiary referral hospital, where patients had reached the hospital when they were already at advanced stage (6). Among multivalve diseases, dual-valve lesion was the most commonly seen across all groups, 39.81% in children and 42.11% in young adults, particularly the combination of mitral and tricuspid regurgitation (MR + TR) in the children's population and the combination of mixed mitral lesion and tricuspid regurgitation (MS + MR + TR) in the young adult population. Combined mitral and tricuspid valve disease is associated with poor survival, with patients more likely to have class III or IV heart failure and reduced functional capacity (22).

Although rheumatic involvement of all four heart valves is rare and often only found on postmortem examination, this study observed a significant occurrence of quadrivalve lesion in the children group (12.96%, *p* = 0.039). Most previous studies, dated back to 15–30 years ago, reported three cases of rheumatic all-valve disease with stenosis, regurgitation, or mixed lesions (23–25).

### Types of Valvular Lesions

Mitral stenosis (either as isolated lesion or in combination) was the predominant mitral valve lesion, affecting 62.57% of young adult RHD patients, consistent with a previous study in Pakistan in which 70% patients were affected (6). In contrast, mitral stenosis was rarely seen in children; rather, mitral regurgitation (isolated or combined) was the ubiquitous valvular presentation (89.81%) of RHD cases. This data was supported by similar findings in other studies in India (8, 26, 27). Mixed mitral valve lesion was more common in young adults (29.24%) than in children (3.70%), which is in agreement with findings in other parts of the world (2, 8). Overall, mitral regurgitation was found to be present in almost every combination of multivalvular lesion.

Tricuspid regurgitation was observed in 74.07% children and 65.49% young adult cases, with tricuspid regurgitation more frequent than mitral valve lesions in young adults, but almost always in combination with mitral valve or in a lower occurrence aortic valve lesions. Our data were much higher than in the previous study, which reported only 5.7% tricuspid regurgitation cases (2). Another study reported tricuspid regurgitation was present in 20.3% of cases (9). Rheumatic involvement of the tricuspid valve is more common when recurrent infections or reactivations have occurred (28). Considering more than half of our patients had involvement of tricuspid valve, it confirmed the much higher prevalence of clinically silent RHD (21.1 per 1,000 people) than clinically manifest RHD (2.7 per 1,000 people) (29). The true incidence of organic tricuspid rheumatic disease might be higher than reported. However, it is important to distinguish organic tricuspid valve involvement from functional tricuspid regurgitation. which is caused by left atrium dilatation, right ventricular dysfunction, and remodeling leading to tricuspid annulus dilatation (22).

Unlike a study by in Zambia Musuku et al. who had no report of affected aortic valve, this study reported an overall 34.68% cases of aortic regurgitation that was slightly more common in children than in young adult (30). Similar data were reported with aortic regurgitation incidence of 47%, and almost always existed in combination (6). Overall, aortic regurgitation was present more commonly than aortic stenosis, with no case of aortic stenosis recorded in children. Similar data were observed in a previous study in Nepal (5).

Damage to the pulmonary valve caused by rheumatic fever is extremely rare, but this study showed involvement of the pulmonary valve in RHD, as pulmonary regurgitation, particularly in children more than in the young adult population; albeit being the least involved and always in combination with other valves. One study in India reported two cases of isolated pulmonary lesion of rheumatic origin in children under 15 years old (31) and one reported pulmonary valve involvement combined with other valves in 2.55% of cases in Nepal (19).

### Difficulties in Long-Term Secondary Prophylaxis

Prevention of recurrent attack or reactivation of RHD is essential in preventing disease morbidity and further complications in the future. Penicillin remains the antibiotic of choice. Oral PMP was given as secondary prophylaxis in all RHD cases. Our data did not include any report on patients' compliance and unexplored follow-up of recurrent attack, since most patients then referred back to their primary or secondary healthcare facilities. However, in clinical practice, we often observed a poor uptake of secondary prophylaxis especially in cases in children.

Inadequate compliance with penicillin therapy was reported in various studies, with non-compliance rate ranging between 4.5 and 70% (2). Oral administration of antibiotics was suggested to be the reason for the high non-compliance rate (2). Considering that, penicillin injection is more desirable, especially for young adults who had low adherence. However, in rural settings, limited access and long travel time to health care facilities, as well as lack of available penicillin therapy, have been documented and have a negative influence on patients' adherence (32). Therefore, it is appropriate to assume that a low compliance rate would also be reported in this study if long-term follow-up was concluded. Patients who took penicillin prophylaxis regularly did not have reactivation of RF, thus emphasizing the importance of adherence to secondary prophylaxis (2). Considering the challenge in compliance rate, benzathine benzylpenicillin injection then should be considered as the drug of choice for secondary prophylaxis, as it would require administration of the drug only once a month. More often it had been noted that RF recurs more frequently in patients on an oral regimen than in those receiving intramuscular benzathine benzylpenicillin, probably due to factors mentioned earlier (2, 16).

### Management of RHD at a Tertiary Care Center

Surgery was frequently the mainstay of treatment, over BMV or medication-only treatment, to alleviate progressive disease caused by chronic rheumatic valvular disease despite adequate and aggressive medical therapy. Surgical treatment in this study was carried out in 59.14% cases in this study, which was higher than previous studies (2, 33). possibly because most patients were referred to the tertiary care late in the course of illness with severely deformed valve and extensive heart damage (3). Surgical intervention can either be valve replacement or valve repair. Valve replacement with a mechanic valve was more common in young adults (56.12%), while in children valve repairment was the most common (79.1%). Surgical valve repair is thought to be the gold standard in heart valve surgery, yet the durability of valve repair might be compromised by the progressive characteristic of rheumatic disease. Ever since, it has been noted from long-term follow-up after surgical valve replacement that the use of a prosthetic ring is a protective factor in the long-term outcome and reduces the risk of reoperation (9). Percutaneous BMV was usually performed in young adults (26.31%). It is the standard first-line therapy for rheumatic mitral stenosis disease, particularly in the absence of mitral insufficiency. However, our study reported BMV was carried out in mitral stenosis and mixed mitral valve lesions with satisfactory results.

Although it had been noted that valvular surgery had its own difficulties regarding the procedure, mechanic valve size, and postoperative complications in a children's population, no postoperative mortality was recorded in this study. Early postoperative mortality was <2% in the young adult group. One patient died after mitral valve replacement due to critical PH, and the other died after failed BMV due to intractable heart failure and critical PH; both were females in their early 20 s. The in-hospital mortality was lower than in the other reports for RHD from low- and middle-income countries, although long-term follow-up after discharge should be noted to show RHD annual mortality.

### Readmission Still Occurred With Various Presentations

Even after administration of appropriate medical therapy, ongoing disability and rehospitalization were still present, emphasizing the need for access to appropriate surgical care. Readmission occurred in fewer than 10% of patients for diverse reasons: reactivation, surgical/valvuloplasty, severe congestive heart failure, and symptomatic atrial fibrillation. Decentralization of services used to diagnosis RHD and creating national registry to record and tracking the disease progression and management may be one of many potential solutions to RHD occurrence in Indonesia or any developing countries.

This study has certain limitations. By obtaining data only from subjects in a single tertiary referral hospital setting, the study is less likely to identify patients with milder manifestation and does not represent the true prevalence and manifestation of RHD in Indonesia.

## Conclusion

RHD remains the major acquired valvular heart disease among children and young adults. Patients with chronic RHD often presented with complications of the disease or with reactivation of RHD. Mitral valve lesion was the predominant lesion, particularly in a dual-valve disease, presenting as mitral regurgitation in children and stenosis in young adults. Tricuspid, aortic, and pulmonary valve involvement was commonly seen with various patterns and combinations. Multivalve lesions indicated an advanced stage of disease. Surgical intervention was carried out, especially in severe cases with low early postoperative mortality. Poor compliance and limited access to secondary prophylaxis remain significant challenges to RF/RHD control programs, especially in rural areas. This study supports the need for more effective preventive methods, early diagnosis, and follow-up care to prevent secondary infection.

## Data Availability Statement

The datasets generated for this study are available on request to the corresponding author.

## Ethics Statement

Written informed consent was obtained from the individual(s), and minor(s)' legal guardian/next of kin, for the publication of any potentially identifiable images or data included in this article.

## Author Contributions

OL: design and conception of the study, data gathering and analysis, writing and final approval of the version to be published. RP, YK, PR, AR, IS, and GH: data gathering. All authors contributed to the article and approved the submitted version.

## Conflict of Interest

The authors declare that the research was conducted in the absence of any commercial or financial relationships that could be construed as a potential conflict of interest.

## References

[1] Myint N Aung N Win M Htut T Ralph A Cooper D. The clinical characteristics of adults with rheumatic heart disease in Yangon, Myanmar: an observational study. PLoS ONE. (2018) 13:e0192880. 10.1371/journal.pone.0192880 29466408 PMC5821331

[2] Joseph N Madi D Kumar G Nelliyanil M Saralaya V Rai S. Clinical spectrum of rheumatic fever and rheumatic heart disease: a 10 year experience in an urban area of south. North Am J Med Sci. (2013) 5:647. 10.4103/1947-2714.122307 24404543 PMC3877438

[3] Sharma P Shakya U KC S Shrestha M Clinical profile and management in children with rheumatic heart disease in a tertiary cardiac care center of Nepal. Nepalese Heart J. (2016) 13:33–6. 10.3126/njh.v13i2.15562

[4] Jacques Cabral T Tantchou Tchoumi J Butera G. Profile of cardiac disease in Cameroon and impact on health care services. Cardiovasc Diagn Ther. (2013) 3:236–43. 10.3978/j.issn.2223-3652.2013.12.05 24400207 PMC3878116

[5] Koirala P Sah R Sharma D Pattern of rheumatic heart disease in patients admitted at tertiary care centre of Nepal. Nepalese Heart J. (2018) 15:29–33. 10.3126/njh.v15i1.19713

[6] Faheem M Hafizullah M Gul A Jan H Khan M Pattern of valvular lesions in rheumatic heart disease. JMPI. (2007) 21:99–103.

[7] Animasahun BA Madise Wobo AD Itiola AY Adekunle MO Kusimo OY Thomas FB. The burden of rheumatic heart disease among children in Lagos: how are we fairing? Pan Afr Med J. (2018) 14:150. 10.11604/pamj.2018.29.150.12603 30050614 PMC6057576

[8] Sani MU Karaye KM Borodo MM. Prevalence and pattern of rheumatic heart disease in the Nigerian savannah: an echocardiographic study. Cardiovasc J Afr. (2007) 18:295–9. 17957324 PMC3975544

[9] Bernal J Pontón A Diaz B Llorca J García I Sarralde J. Combined mitral and tricuspid valve repair in rheumatic valve disease. Circulation. (2010) 121:1934–40. 10.1161/CIRCULATIONAHA.109.894873 20404254

[10] Watkins D Beaton A Carapetis J Karthikeyan G Mayosi B Wyber R. Rheumatic heart disease worldwide. J Am Coll Cardiol. (2018) 72:1397–416. 10.1016/j.jacc.2018.06.063 30213333

[11] Carapetis J. Rheumatic heart disease in Asia. Circulation. (2008) 118:2748–53. 10.1161/CIRCULATIONAHA.108.774307 19106399

[12] Zühlke L Beaton A Engel M Hugo-Hamman C Karthikeyan G Katzenellenbogen J. Group A Streptococcus, acute rheumatic fever and rheumatic heart disease: epidemiology and clinical considerations. Curr Treat Options Cardiovasc Med. (2017) 19:15. 10.1007/s11936-017-0513-y 28285457 PMC5346434

[13] Gewitz M Baltimore R Tani L Sable C Shulman S Carapetis J Revision of the Jones criteria for the diagnosis of acute Rheumatic fever in the era of Doppler echocardiography. Circulation. (2015) 131:1806–18. 10.1161/CIR.0000000000000205 25908771

[14] Boyarchuk O Hariyan T Kovalchuk T Clinical features of rheumatic heart disease in children and adults in Western Ukraine. Bangladesh J Med Sci. (2019) 18:87–93. 10.3329/bjms.v18i1.39556

[15] Kadir IS Barker TA Clarke B Denley H Grotte GJ. Recurrent acute rheumatic fever: a forgotten diagnosis? Ann Thorac Surg. (2004) 78:699–701 10.1016/S0003-4975(03)01376-6 15276555

[16] World Health Organization Rheumatic Fever and Rheumatic Heart Disease: Report of a WHO Expert Consultation. (2001). Geneva: World Health Organization (2004).

[17] Rayamajhi A Sharma D Shakya U. Clinical, laboratory and echocardiographic profile of acute rheumatic fever in Nepali children. Annals Tropical Paediatrics. (2007) 27:169–77. 10.1179/146532807X220271 17716444

[18] Manjunath CN Srinivas P Ravindranath KS Dhanalakshmi C. Incidence and patterns of valvular heart disease in a tertiary care high-volume cardiac center: a single center experience. Indian Heart J. (2014) 66:320–6. 10.1016/j.ihj.2014.03.010 24973838 PMC4121759

[19] Laudari S Subramanyam G. A study of spectrum of rheumatic heart disease in a tertiary care hospital in Central Nepal. Int J Cardiol Heart Vasc. (2017) 15:26–30. 10.1016/j.ijcha.2017.03.007 28616570 PMC5458122

[20] Alkhalifa MS Ibrahim SA Osman SH. Pattern and severity of rheumatic valvular lesions in children in Khartoum, Sudan. East Mediterr Health J. (2008) 14:1015–21. 19161072

[21] Reményi B Wilson N Steer A Ferreira B Kado J Kumar K. World Heart Federation criteria for echocardiographic diagnosis of rheumatic heart disease–an evidence-based guideline. Nat Rev Cardiol. (2012) 9:297–309. 10.1038/nrcardio.2012.7 22371105 PMC5523449

[22] Shiran A Sagie A. Tricuspid regurgitation in mitral valve disease. J Am Coll Cardiol. (2009) 53:401–8. 10.1016/j.jacc.2008.09.048 19179197

[23] Jai Shankar K Jaiswal PK Cherian KM. Rheumatic involvement of all four cardiac valves. Heart. (2005) 91:e50. 10.1136/hrt.2005.060509 15894751 PMC1768928

[24] Bandin MA Vargas Barron J Keirns C Romero-Cardenas A Villegas M Buendia A. Echocardiographic diagnosis of rheumatic cardiopathy affecting all four cardiac valves. Am Heart J. (1990) 120:1004–7. 10.1016/0002-8703(90)90230-U 2220528

[25] Krishnamoorthy KM. Rheumatic stenosis of all four valves. Texas Heart Inst J. (2002) 29:224–5. 12224732 PMC124768

[26] Ravisha MS Tullu MS Kamat JR. Rheumatic fever and rheumatic heart disease: clinical profile of 550 cases in India. Arch Med Res. (2003) 34:382–7. 10.1016/S0188-4409(03)00072-9 14602504

[27] Negi PC Kanwar A Chauhan R Asotra S Thakur JS Bhardwaj AK. Epidemiological trends of RF/RHD in school children of Shimla in north India. Ind J Med Res. (2013) 137:1121–7. 23852293 PMC3734717

[28] Dass C Kanmanthareddy A. Rheumatic Heart Disease. StatPearls. (2019). Available online at: https://www.ncbi.nlm.nih.gov/books/NBK538286/ (accessed March 12, 2020).

[29] Rothenbühler M O'Sullivan CJ Stortecky S Stefanini GG Spitzer E Estill J. Active surveillance for rheumatic heart disease in endemic regions: a systematic review and meta-analysis of prevalence among children and adolescents. Lancet Glob Health. (2014) 2:e717–26. 10.1016/S2214-109X(14)70310-9 25433627

[30] Musuku J Engel ME Musonda P Lungu JC Machila E Schwaninger S. Prevalence of rheumatic heart disease in Zambian school children. BMC Cardiovasc Disord. (2018) 18:135. 10.1186/s12872-018-0871-8 29969998 PMC6029054

[31] Muthiah R Isolated rheumatic pulmonary valve disease—case reports. Case Reports Clin Med. (2016) 5:207–15. 10.4236/crcm.2016.56039

[32] Kevat PM Reeves BM Ruben AR Gunnarsson R. Adherence to secondary prophylaxis for acute rheumatic fever and rheumatic heart disease: a systematic review. Curr Cardiol Rev. (2017) 13:155–66. 10.2174/1573403X13666170116120828 28093988 PMC5452151

[33] Bitar FF Hayek P Obeid M Gharzeddine W Mikati M Dbaibo GS. Rheumatic fever in children: a 15-year experience in a developing country. Pediatr Cardiol. (2000) 21:119–22. 10.1007/s002469910017 10754079

